# Genome-Wide Modeling of Transcription Preinitiation Complex Disassembly Mechanisms using ChIP-chip Data

**DOI:** 10.1371/journal.pcbi.1000733

**Published:** 2010-04-01

**Authors:** Eric Samorodnitsky, B. Franklin Pugh

**Affiliations:** 1 Center for Eukaryotic Gene Regulation, The Pennsylvania State University, University Park, Pennsylvania, United States of America; 2 Center for Comparative Genomics and Bioinformatics, The Pennsylvania State University, University Park, Pennsylvania, United States of America; 3 Department of Biochemistry and Molecular Biology, North Frear Laboratory, The Pennsylvania State University, University Park, Pennsylvania, United States of America; The Hebrew University, Israel

## Abstract

Apparent occupancy levels of proteins bound to DNA in vivo can now be routinely measured on a genomic scale. A challenge in relating these occupancy levels to assembly mechanisms that are defined with biochemically isolated components lies in the veracity of assumptions made regarding the in vivo system. Assumptions regarding behavior of molecules in vivo can neither be proven true nor false, and thus is necessarily subjective. Nevertheless, within those confines, connecting in vivo protein-DNA interaction observations with defined biochemical mechanisms is an important step towards fully defining and understanding assembly/disassembly mechanisms in vivo. To this end, we have developed a computational program PathCom that models in vivo protein-DNA occupancy data as biochemical mechanisms under the assumption that occupancy levels can be related to binding duration and explicitly defined assembly/disassembly reactions. We exemplify the process with the assembly of the general transcription factors (TBP, TFIIB, TFIIE, TFIIF, TFIIH, and RNA polymerase II) at the genes of the budding yeast *Saccharomyces*. Within the assumption inherent in the system our modeling suggests that TBP occupancy at promoters is rather transient compared to other general factors, despite the importance of TBP in nucleating assembly of the preinitiation complex. PathCom is suitable for modeling any assembly/disassembly pathway, given that all the proteins (or species) come together to form a complex.

## Author Summary

For proper cell function, cells need to precisely coordinate the expression of their genes on their DNA at precise times. In order to better understand how the cell works, it is important to understand how, when, and why a cell needs to turn on or off certain genes at certain times. In order to assist the cell to properly express its genes, there are hundreds of proteins that can bind and access DNA. Each protein has a unique function and these proteins assemble together into a very large complex to turn on genes. The assembly of these proteins has defined to some extent, however the whole process of assembly and disassembly of this complex in the cell is still poorly understood. In our modeling analysis, we have attempted to utilize genome-wide binding data to better understand how the transcription machinery that “reads” genes might disassemble, in light of what is known about the assembly process. This knowledge helps us better understand how cells coordinate their on/off-switching of their genes.

## Introduction

Eukaryotic genes are thought to be regulated by hundreds of proteins that assemble into pre-initiation complexes (PIC's) at promoters using an ordered pathway. One aspect of the PIC assembly pathway involves the recruitment of the general transcription factors (GTF's), such as TBP and TFIIB, by sequence-specific activators. TBP and TFIIB then contribute to the recruitment of RNA polymerase II (pol II) and other GTF's, which eventually start transcription.

A fundamental question concerning our understanding of gene regulation is the extent to which each assembly and disassembly step is distinct at every gene in a genome. Is the traditional biochemical view that TBP “locks in” or commits to a promoter, and in a recurring manner nucleates PIC formation valid in vivo? And is the PIC disassembly process in vivo, simply the reverse of the assembly process? Parts of the assembly/disassembly pathway have been rigorously defined in vitro with a few purified proteins and DNA, and this has provided us with our current parsimonious view of PIC regulation [1],[2],[3],[4]. In no case have assembly or disassembly reactions been reconstituted in a way that fully recapitulates the physiological setting (presence of sequence-specific regulators, coactivators, specifically positioned nucleosomes, chromatin regulators, GTFs, etc) at every gene, and so these questions remain open, in regards to the extent to which in vitro defined reactions mimic the in vivo events occurring throughout a genome. The answer to this question is not readily addressed in vivo, since reactions are not as definable nor quantifiable as in vitro biochemical reactions with purified components. Nonetheless, assays do exist for measuring relative levels of protein•DNA complex formation in vivo, and so mechanistic inferences will be sought.

The goal here is to evaluate in vivo occupancy data in light of biochemical mechanisms that are intended to reflect the corresponding in vivo reaction. The extent of biological insight is predicated on rather subjective assessments of the assumptions associated with interpretation of in vivo data. Within the context of declared constraints and assumptions, we propose a means to model in vivo protein-DNA occupancy data, so as to better integrate and conceptualize massive genomic datasets. This study is focused on the means of such modeling and the assumptions inherent in the data, using specific examples of PIC assembly.

Currently, perhaps the most widely used assay to measure the occupancy of proteins at genes in vivo is the chromatin immunoprecipitation assay (ChIP). In ChIP, proteins are crosslinked to DNA, the protein is then purified, and the bound DNA identified either through directed PCR or through genome-wide detection platforms (ChIP-chip and ChIP-seq). In this way, for example, the relative occupancy level of TBP, TFIIB, pol II, and many other proteins at every promoter in the genome in a population of cells can be assayed.

Recent studies using differential ChIP and photobleaching experiments have provided compelling evidence for a dynamic state of PIC components in living cells [5],[6],[7]. Therefore, it is now within a conceptual framework to expect factors like RNA polymerase II, TBP, and other GTFs to undergo multiple assembly and disassembly cycles at promoters for each productive transcription event, rather than the traditional simple view that GTF's remain locked in place during multiple transcription cycles.

The existence and origins of distinct occupancy levels of PIC components on genes has not been systematically explored, and thus is the impetus for conducting the modeling studies described here. Differential occupancy patterns for the GTFs have been described [8], and may be caused by gene-specific regulators that influence the recruitment or retention of specific general transcription factors (among other proteins), and thus assembly/disassembly mechanisms might differ from gene to gene (or sets of genes). Here, we attempt to utilize ChIP-chip binding information gleaned at every promoter in the yeast genome to either plausibly infer or exclude PIC assembly/disassembly mechanisms. The major limitation in any such approach is that the number of permutations of possible assembly/disassembly mechanisms exceeds the amount of data available to constrain such mechanisms. In other words, occupancy data, alone, is insufficient to uniquely specify an ordered PIC assembly and disassembly pathway. Imposition of additional constraints (or assumptions), such as predefining either the assembly (or disassembly) pathway, might however eliminate certain dissociation (or association) mechanisms as incompatible with the data, and thus serves the purpose of plausibly excluding mechanisms rather than uniquely identifying a mechanism.

Here, we develop a ChIP modeling program, termed PathCom, in the context of a fixed PIC assembly pathway to infer allowable dissociation mechanisms. We validate the simulation using an existing chemical kinetics simulator COPASI [9]. Within the declared constraints, we discern the compatibility of different PIC disassembly mechanisms at nearly every transcriptionally-active gene in the yeast genome with existing ChIP-chip occupancy data.

## Results

### Genome-wide occupancy modeling of two factors

The overall goal here is to inter-relate ChIP in vivo occupancy data with biochemical assembly/disassembly mechanisms, in a way that attempts to support or dispute such mechanisms. Such inter-relationships can be complex when one considers that hundreds of proteins are involved in transcriptional regulation. Therefore, we start by modeling only two factors (the GTF's TBP and TFIIB), and increase complexity by adding more GTFs one at a time up to six factors. While we focus on PIC assembly/disassembly mechanisms on a genomic scale, any number of factors and combination of assembly/disassembly steps in gene regulation may be considered, given that all proteins (or species) come together to form a complex.

**Figure 1:**
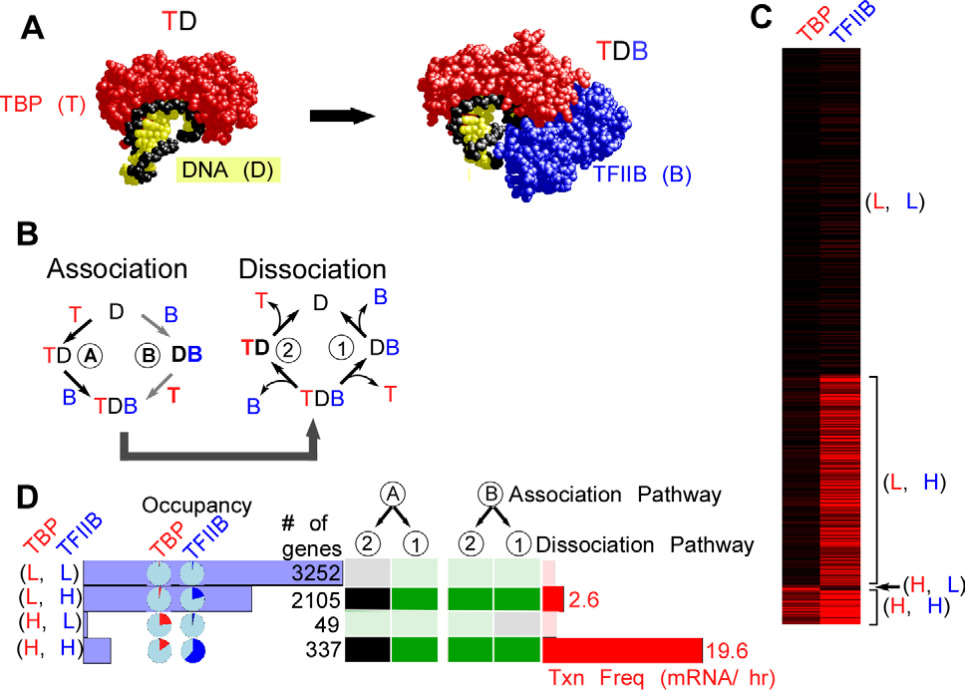
Two factor (TBP and TFIIB) modeling of genome-wide ChIP occupancy data. **A**, Crystal structure models of a TBP•TATA complex [11],[12] and a TBP•DNA•TFIIB complex [13]. **B**, Alternative association/dissociations mechanisms of TBP (T), TFIIB (B), and DNA (D). **C**, Cluster-plot showing the occupancies of TBP and TFIIB at individual genes (rows), scaled from 0% (black) to 100% (red). **D**, Shown are data for four gene groups defined by their high (H) or low (L) factor occupancy level. For example, (L,H) group contains 2105 genes having low TBP occupancy (<10% of the maximum) and high TFIIB occupancy (>10% of the maximum). Horizontal blue bar graphs indicate the number of genes in each of the four groups. Pie charts indicate the median occupancy level (red for TBP and blue for TFIIB) for the indicated gene groups. The table of black/green squares represents PathCom output for incompatibility (black) or compatibility (green) with the indicated mechanism (described in panel B). Median transcription frequencies for genes in each group are shown as horizontal red bars [34].

TBP (T) binds to DNA (D) to form a protein-DNA (TD) complex, and in the presence of TFIIB (B) form a TDB ternary complex (**Figure 1A**) [10],[11],[12]. In the presence of sufficient levels of these proteins, their DNA occupancy level will vary from 0% to 100% as dictated by the context of each promoter. In principle, there are two pathways by which TBP and TFIIB assemble step-wise onto DNA (**Figure 1B**) [13]: A) TBP binds to DNA, then TFIIB binds; or B) TFIIB binds DNA first, then TBP. Their reversal constitutes two pathways for dissociation.

The constant availability of energy to drive directional processes allows the pairing of any association and dissociation mechanism. Consequently, there are four paths by which an in vivo occupancy level is achieved for a two-component reaction. The availability of only two experimental constraints (TBP and TFIIB occupancy levels on DNA) is insufficient to specify the predominant association and dissociation pathways. In the absence of a necessary additional experimental constraint, we created a hypothetical constraint for the purposes of modeling, in which we eliminated all but one association pathway. That allowed us to evaluate the two possible dissociation pathways. The reciprocal modeling could also be done, by eliminating all but one dissociation mechanism. Since the purpose of this study is to demonstrate how the modeling works and to discuss its assumptions, caveats, and utility, we illustrate the process using a single association pathway that has good experimental support and model all possible dissociation pathways.

Biochemical [1] and crystallographic [13] evidence shows that TBP binds DNA first, followed by TFIIB, which makes cooperative contacts with both TBP and the DNA (**Figure 1A**). On this basis, we fixed assembly pathway “A” (**Figure 1B**), which sufficiently constrains the system so that measured TBP and TFIIB occupancy levels can distinguish between the two dissociation pathways, “1” and “2”. In this context, dissociation pathway “1” allows either TBP or TFIIB occupancy to be greater than the other, but pathway 2 is only plausible if TBP occupancy is greater.

Using published genomic datasets of TBP and TFIIB occupancy [14], we modeled four groups of genes, each having either a high (H) or low (L) experimentally measured level of TBP and TFIIB (**Figure 1C**, and **Figure S1**). These occupancy levels were reproducible and verified by a second data source (Affymetrix high density tiling arrays) also present in the previous study (**Figure S2**) [14]. We chose four subdivisions so as to separately consider different types of occupancy patterns. In principle, each gene could be treated independently. However, grouping of similarly behaving genes had the advantage of creating more robust occupancy values that are based upon hundreds of measurements, rather than just one. Aggregating the data dampened the variability caused by gene-specific differences in crosslinking efficiency and detection. It also served to identify predominant occupancy patterns that might reveal underlying themes in gene regulation. One limitation of such grouping is that it assumes a single underlying mechanism exists for an individual gene and for an entire group of genes, which may be unlikely in detail but reasonable for purposes of demonstration.

To compare occupancy levels between proteins, it was necessary to place them on the same scale. We achieved this by scaling ChIP occupancy values (fold over background) for each factor from 0% to 100%. Our rationale, assumptions, and method for doing this are described in the Methods section.

**Figure 1D** shows a cluster-plot of the genes with their TBP and TFIIB percent occupancies. Since the “(L, L)” group (**Figure 1F**) had low levels of both factors, TBP and TFIIB did not substantially occupy these genes. Consequently, modeling would not be informative for this group, and thus was not examined further. In addition, the “(H, L)” group comprised <1% of all genes, and so it too was not examined further. For the remaining two groups, TFIIB occupancy was greater than TBP occupancy. When assembly pathway A was fixed, in which TFIIB assembles last, then the observed higher level of TFIIB occupancy over TBP can only be accommodated by a situation where TFIIB dissociates last. Thus, for both groups ((L, H) and (H, H)), the data reject dissociation pathway 2 (TFIIB dissociates first) and accept pathway 1. These outcomes are illustrated in **Figure 1D**, by the black (incompatible) and green (compatible) squares. Note that when the alternative assembly pathway B is fixed, both dissociation pathways were compatible. This simple case illustrated how different starting assumptions (assembly pathway A vs B) resulted in a different set of compatibility outcomes.

From this analysis, several insights were obtained: 1) Some occupancy levels simply do not distinguish among mechanisms. 2) In contrast to the simplified in vitro derived biochemical mechanism, TFIIB might remain at most promoters after TBP has dissociated (although TFIIB may nevertheless be dynamic). How TFIIB does so is a matter of speculation that the data do not address.

Based upon known TBP/TFIIB/DNA biochemical interactions, the notion that TFIIB might dissociate after TBP would seem untenable. However, the additional complexity that exists in vivo might accommodate such a mechanism if other proteins not explicitly defined in this model retained TFIIB at the promoter, after TBP had dissociated. TFIIB engages pol II at promoters via specific interactions [15],[16],[17]. Pol II tightly associates with DNA in an “open” promoter complex [18],[19], and tends to accumulate at the 5′ ends of genes [14],[20],[21],[22]. If an active mechanism removes TBP, such as through the well-established ATP-dependent mechanism of Mot1 [23], then TFIIB might remain on promoter DNA via pol II and in the absence of TBP.

### Development of PathCom to model three factor occupancy

**Figure 2:**
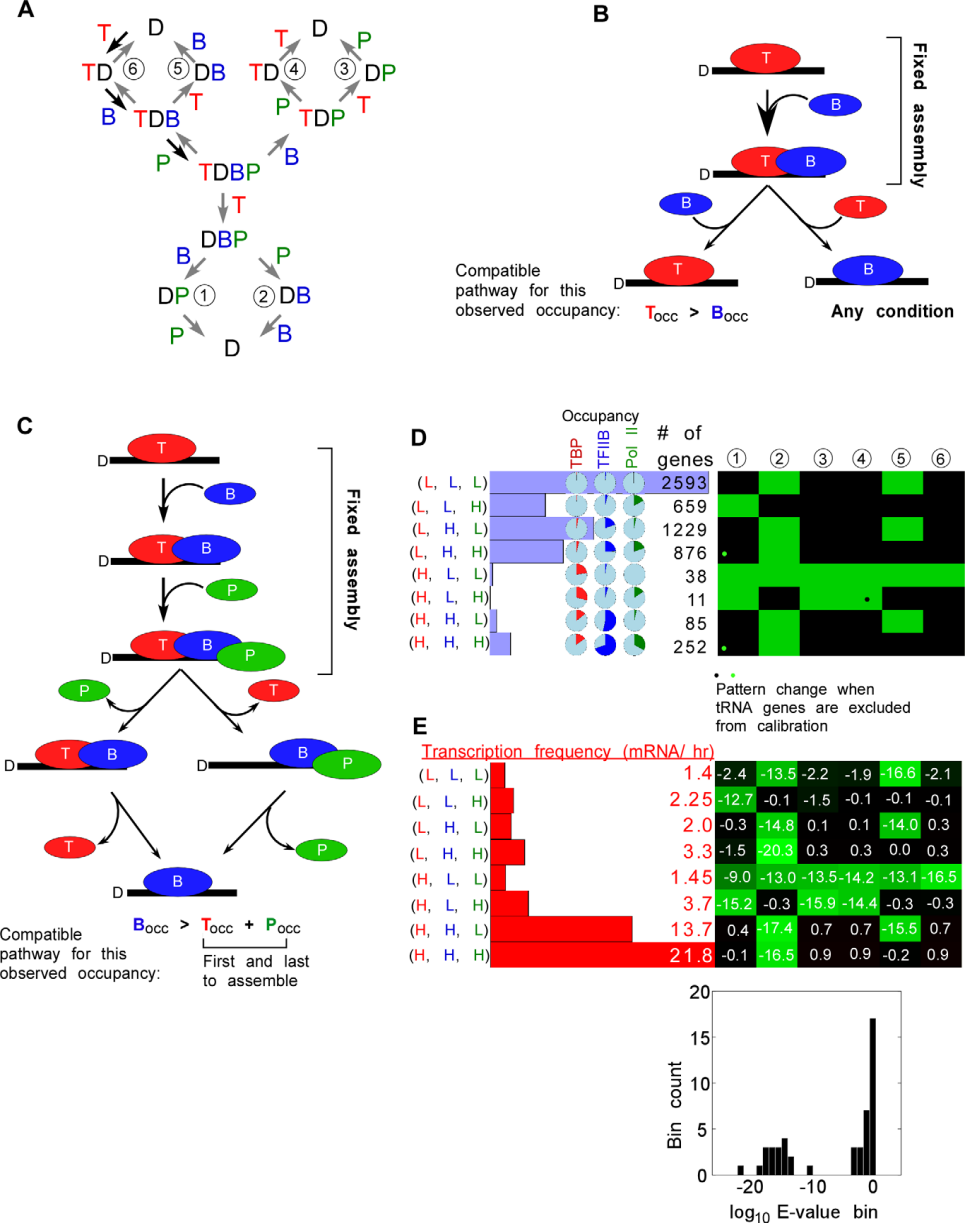
Three factor (TBP, TFIIB, and Pol II) modeling of genome-wide ChIP occupancy data. **A**, Alternative dissociation pathways modeled are shown. The fixed assembly pathway is illustrated with the black arrows. **B**, The first rule of compatibility is pictorially represented. Note that, given the assembly pathway, the disassembly pathway on the left requires TBP occupancy to be greater than TFIIB occupancy, whereas the disassembly pathway on the right can support either TBP or TFIIB occupancy being greater. **C**, The second rule of compatibility is illustrated. If TFIIB occupancy is greater than the combined occupancy of TBP and pol II, then only the disassembly pathways shown will work. **D**, Membership bar graphs, occupancy pie graphs, and the PathCom compatibility cluster plot are described in Figure 1D. TBP binding was found to be highest at tRNA genes and we wanted to assess if removing these genes would substantially alter the compatibility pattern. We found that only 3 of 48 tests were affected (indicated by opposing green and black dots). Note that given the rules of compatibility, some columns (mechanisms) are more constrained than others. **E**, Transcription frequency bar graphs for each group is shown, along side the COPASI compatibility cluster plot. Below that, is a histogram showing the distribution of log_10_ E-values. It is clearly bimodal. The group of bars at the very left represent incompatible E-values, while the rest of them represent compatible E-values.

Towards our goal of modeling the assemblage of many proteins, we next consider a three-factor assemblage. The interaction of TFIIB with pol II (P) and TBP is structurally and biochemically well defined [13],[15]. As in the two-step modeling, based upon biochemical precedent, we constrain the system to the following assembly pathway: TBP → TFIIB → pol II (**Figure 2A**, black arrows). Since there are three factors, there are six possible dissociation pathways. Modeling three factors through six mechanisms for eight groups of genes became conceptually challenging to work through in the intuitive manner described for two factors. However, we determined that the plausibility of any mechanism could be evaluated by two basic rules:

**Rule 1: Does the mechanism make it unconditional that one protein's occupancy level must be greater than another?** For example, in the two factor mechanism, if TFIIB enters last and leaves first (**Figure 2B**, left path), then such a mechanism requires that TFIIB occupancy be less than TBP occupancy. On the other hand, if TFIIB leaves last (**Figure 2B**, right path), then such a mechanism allows both TBP and TFIIB to occupy the DNA independent of the other. This mechanism will therefore accommodate any occupancy levels observed for these proteins.

**Rule 2: Does the occupancy of one protein, other than the first and last proteins to assemble, have an occupancy level greater than the summed occupancy of any previously-associating protein and any subsequently-associating protein? If so, does the mechanism give the possibility that the protein's occupancy is greater than the combined occupancies of these two other proteins?** This rule is applicable towards modeling of more than two factors. When this condition is met, then the protein must at some point occupy DNA without the other two proteins, and thus must be the last of the three to dissociate (but not necessarily the last to dissociate overall if the mechanism has more than three proteins). When iterated over all factors in a mechanism, this rule determines the allowable orders of dissociation. For example, consider a fixed assembly order with TBP first, then TFIIB, then pol II (**Figure 2C**): If TFIIB occupancy is greater than the sum of TBP and pol II occupancy, then only those dissociation mechanisms that have TFIIB dissociate last are compatible. If this condition is not true, then any dissociation mechanism can be accommodated by this rule, including the ones having TFIIB dissociate last (but some might be disallowed in the context of rule 1).

These two rules, together, determine which dissociation mechanisms will be compatible with the data given an assumed association pathway. Note that depending on the actual percent occupancies, these rules will have varying effectiveness in narrowing down the dissociation mechanisms. If the rank order of observed occupancy is the same as the order of association, then all dissociation mechanisms will work.

We transformed these queries into a program termed PathCom (short for Pathway Compatibility), which was used to generate the compatibility chart in **Figure 2D** (green  =  compatible, black  =  incompatible). This software is available in Protocol S1 and Protocol S2 for Windows and Mac users, respectively. Using the rationale from the two-step model, we generated eight groups of genes corresponding to either high or low occupancy of each of the factors (**Figure 2D**).

We sought to validate the approach taken by PathCom, to ensure that it reflected enzymological concepts for which this modeling attempts to emulate. Our validation employed COPASI, a freely available program that simulates biochemical kinetics [9]. Reaction mechanisms and concentrations (the latter equivalent to the occupancy levels described here) represent input parameters. For each mechanism and each group of genes, COPASI iteratively “guesses and checks” in an attempt to find a set of rate constants that delivers the observed occupancy levels for TBP, TFIIB, and pol II. It then reports a goodness-of-fit by measuring the square difference between the observed and the optimized occupancies, reporting this as an E-value (see Methods).

To maximize the parameter search space and avoid local minima, COPASI imposes some randomness in moving through the decision-making process. Since the system is under-constrained and randomness is involved, each repeated modeling run converges on a different solution for each mechanism (i.e., many different combinations of rate constant values can produce the observed occupancy levels, if a solution can be found). The values of the underlying rate constants generated by the Parameter Estimator in COPASI are not meaningful; rather the resulting E-value provides a quantitative measure of the suitability of a mechanism to fit the data. Re-running COPASI on the same dataset returns essentially the same E value (not shown). Thus, COPASI provides a robust means of evaluating alternative mechanisms and validating PathCom.

**Figure 2D** shows the compatibility findings of all eight possible clusters using three factors against the six possible dissociation mechanisms using PathCom. **Figure 2E** shows the corresponding log_10_ E-value assessments using COPASI. In all cases, the COPASI-reported E-values matched the Boolean decisions made by PathCom (compare **Figure 2D** and **E**). Log_10_ E-values generated by COPASI were bimodal (**Figure 2E**, bottom bar graph), providing a demarcation between compatible and incompatible outcomes. Thus, the simplified Boolean process associated with PathCom was validated by a kinetic mechanism simulator (COPASI).

Importantly, the analysis indicates that given a fixed association mechanism, there are a limited number of dissociation mechanisms (green squares in **Figure 2D**) that can account for the observed occupancy data. Fixing different association pathways generates different mechanism compatibility patterns (**Figure S3**). In **Figure 2D**, clusters of genes that had very few members (e.g., (H, L, L) and (H, L, H)), or had very low occupancy of all tested factors (e.g. (L, L, L)) may not be particularly robust, and thus less reliably interpreted. For the remaining clusters, one to two mechanisms were found to be compatible. A common theme was that TBP dissociated first, then pol II, and then TFIIB, which was consistent with the conclusions drawn from the two-factor assembly analysis described above.

In principle, dissociation of pol II may proceed via removal into the bulk nucleoplasm and/or translocation down the DNA upon transcription, where ChIP occupancy would not be detected by microarray probes at the 5′ ends of genes. Consistent with the latter possibility, high transcription frequencies are observed at the (H, H, L) set, which has high TBP and TFIIB occupancy but relatively low occupancy of pol II (**Figure 2C**). These genes are also enriched with pol II in the body of the gene (not shown).

The suggestion that TFIIB dissociates after both TBP and pol II dissociation is consistent with some reports in the literature [24], and suggests that perhaps other factors retain TFIIB at promoters in the absence of TBP and pol II. TFIIB and TFIIF are known to interact with each other [25], and potentially with activators [24],[26],[27],[28].

We further examined the plausibility that TBP might not be fully bound at “high” occupancy promoters by looking at experimentally determined “digital footprints” of TBP bound at those promoters having the highest TBP occupancy (**Figure S4**) [29]. Indeed, in all cases, no TBP footprint was detected over the TATA box, which is consistent with the notion that TBP does not fully occupy even its most highly occupied sites.

Groups of genes that had very few members (e.g., (H, L, L) and (H, L, H)), or had very low occupancy of all tested factors (e.g. (L, L, L)) are expected to have higher variation, and thus less reliably interpreted. Therefore, these groups were not examined further. For the remaining groups, one to two mechanisms were found to be compatible. A common theme was that TBP dissociated first, then pol II, and then TFIIB, which was consistent with the conclusions drawn from the two-factor assembly analysis described above.

### Four, five and six factor PIC assembly

As more factors were added to the modeling, and genes grouped according to low or high occupancy levels of each protein, the number of possible groups grew exponentially (2^n^, where is the number of modeled proteins). Consequently, membership in each group diminished, some to negligible levels. Those with negligible membership did not represent predominant patterns and may have arisen by chance as a consequence of noisy occupancy levels. Therefore, we combined groups of genes that lacked a viable membership level (see Methods for membership criteria).

**Figure 3:**
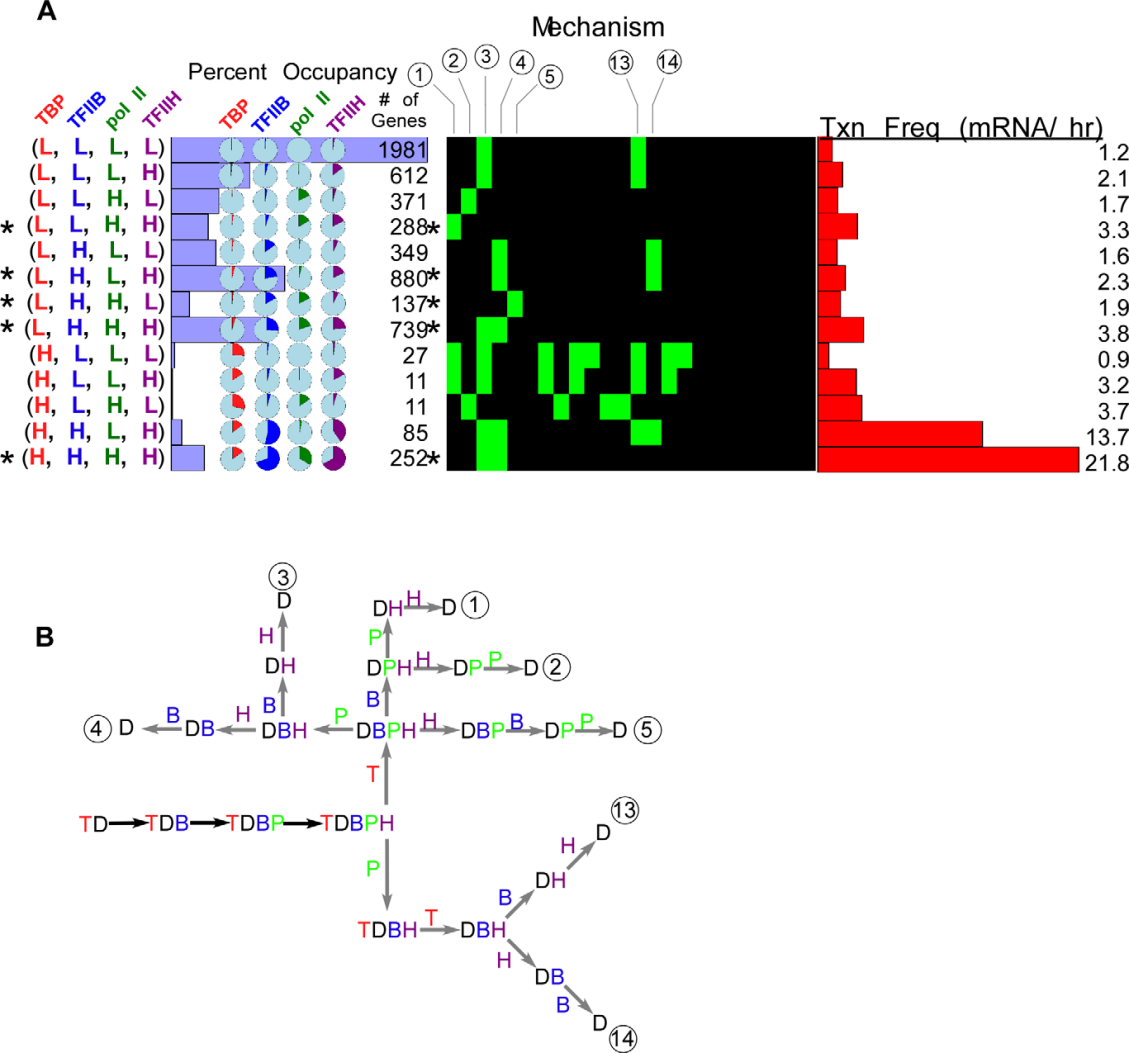
Four factor (TBP, TFIIB, Pol II, and TFIIH) modeling of genome-wide ChIP occupancy data. **A–B**, See Figures 1 and 2 for panel descriptions.

Using the in vitro model for PIC assembly, we next added TFIIH (H) to the mechanism: TBP → TFIIB → pol II→ TFIIH. This mechanism is applicable even if pol II and TFIIH were entering together. As shown in **Figure 3A**, the groups with the highest membership of genes included those with low TBP occupancy levels, and either low or high levels of the other GTFs (indicated by asterisks for gene groups that had at least two high occupancy GTFs). A group having high levels of all GTFs predominated among those groups having high TBP occupancy, denoted (H, H, H, H). In the context of the modeled assembly pathway, these results suggest that TBP is removed from most measured genes before the other GTFs, except in cases where PIC assembly is maximal. The latter could be interpreted to reflect continuous reloading of TBP, which has recently been shown to be fairly dynamic [6],[7]. Our modeling studies with PathCom suggest that the most plausible mechanisms for gene groups with abundant membership and at least two high abundance GTFs include early TBP dissociation (**Figure 3B**). However for one abundant gene set (L, H, L, H), the data are also compatible with an early dissociation of pol II followed by TBP (or simultaneous with it) (**Figure 3B**, dissociation mechanisms 13 and 14).

In the four-factor mechanism, groups having a relatively large gene membership typically were limited to being compatible with only one or two of the 24 theoretically possible dissociation mechanisms (**Figure 3A**, compatibility chart). Thus, the modeling of more factors increased the number of potential mechanisms in a factorial relationship (n!) with the number (n) of proteins being modeled. However, the number of plausible mechanisms remained largely fixed at one to two, with a few exceptions.

**Figure 4:**
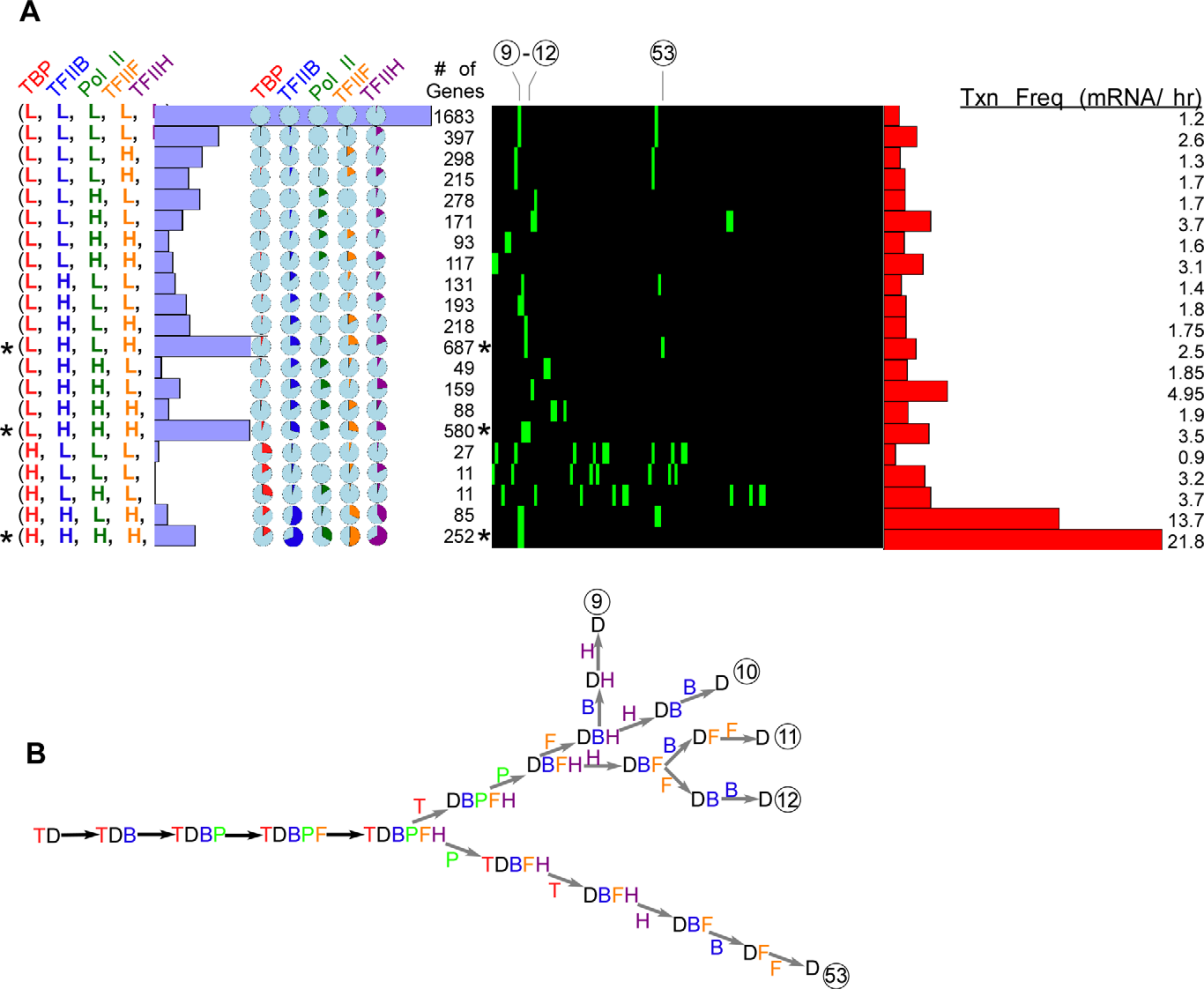
Five factor (TBP, TFIIB, Pol II, TFIIF, and TFIIH) modeling of genome-wide ChIP occupancy data. **A–B**, See Figures 1 and 2 for panel descriptions.

**Figure 5:**
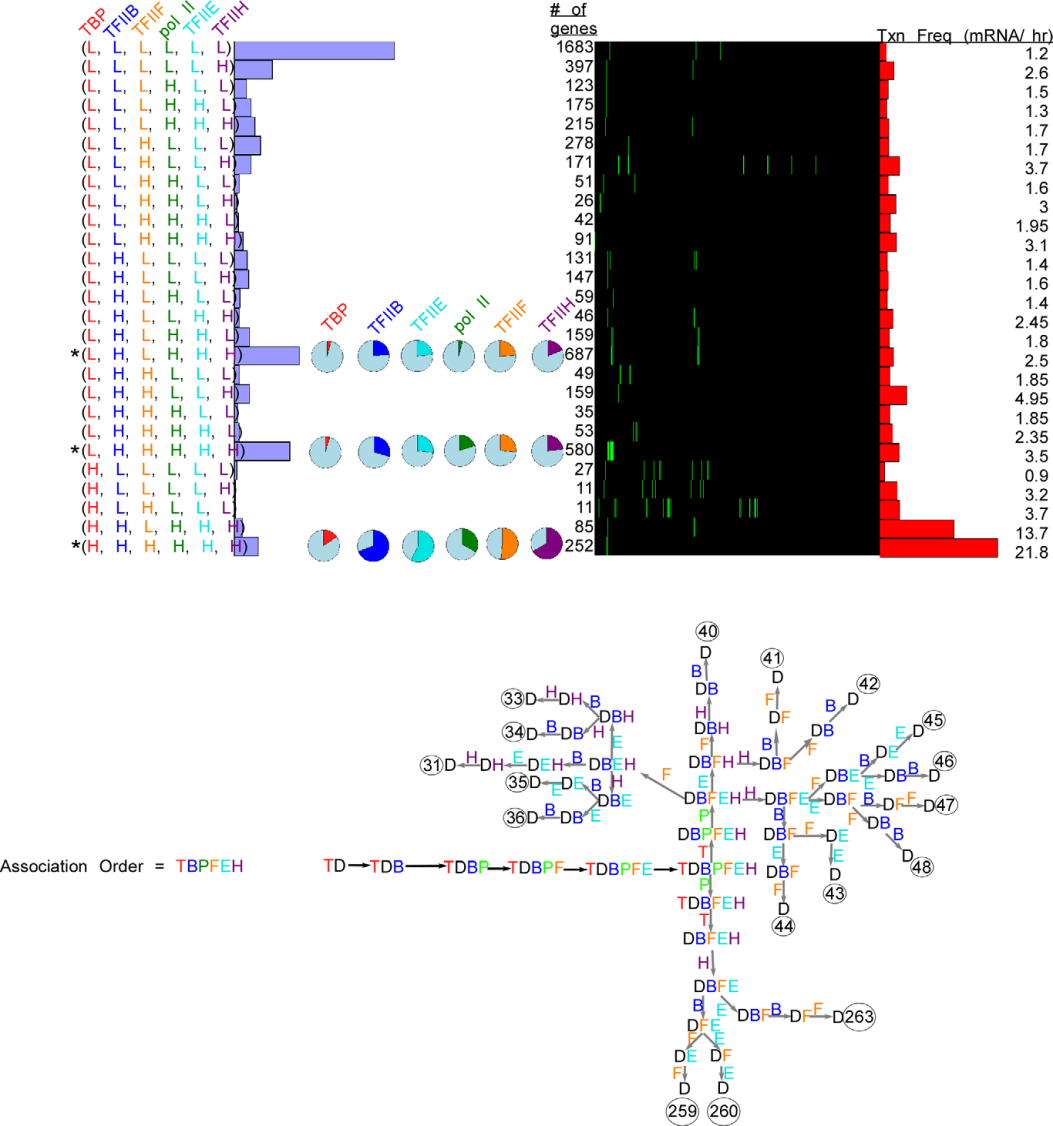
Six factor (TBP, TFIIB, Pol II, TFIIF, TFIIE, and TFIIH) modeling of genome-wide ChIP occupancy data using the assembly pathway TBP → TFIIB → pol II→ TFIIF → TFIIE → TFIIH. See Figures 1 and 2 for panel descriptions.

**Figure 6:**
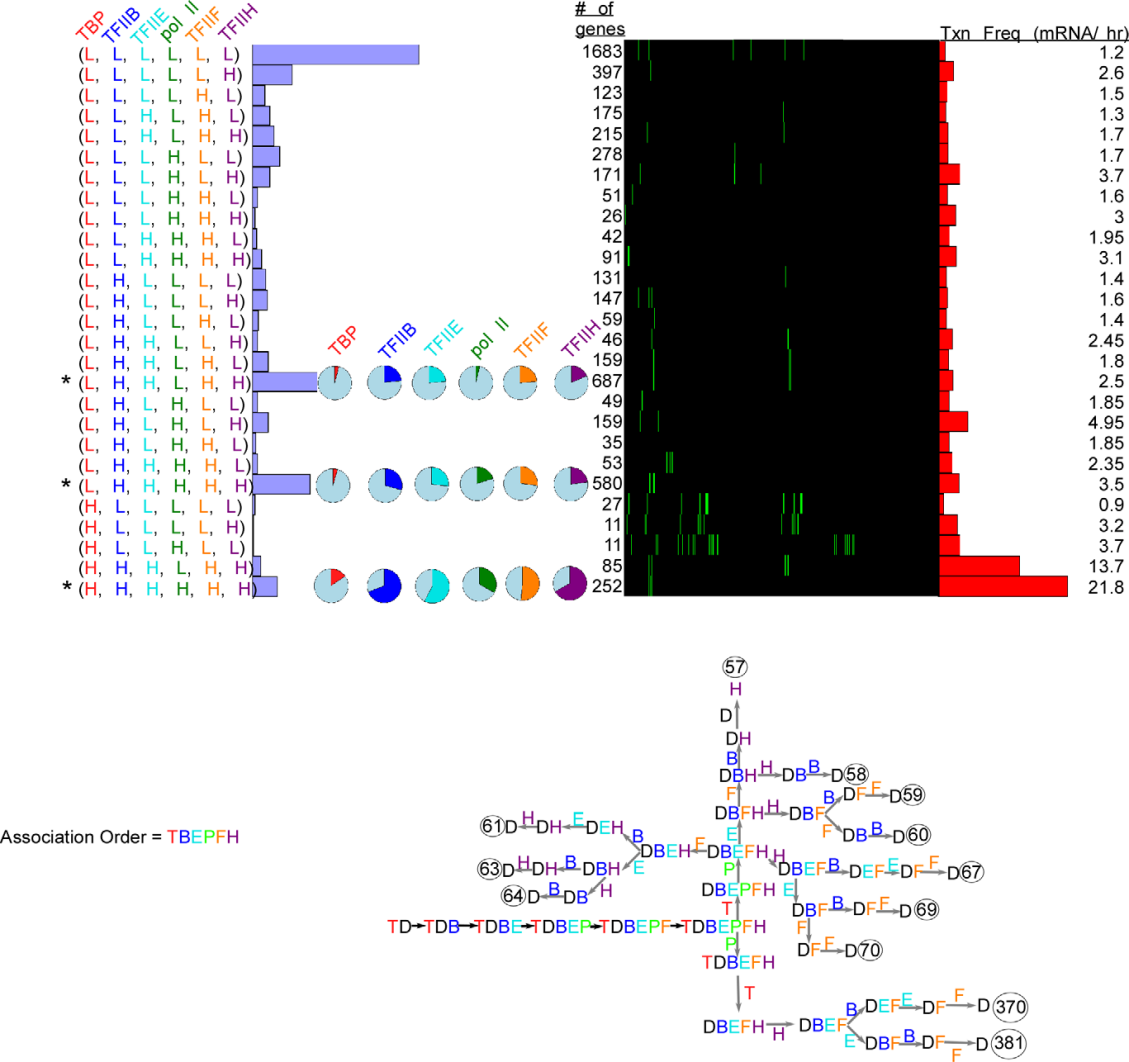
This figure is the same as Figure 5, except the assembly pathway is TBP → TFIIB → TFIIE → pol II → TFIIF → TFIIH.

We next added TFIIF (F) (**Figure 4**) and TFIIE (E) (**Figure 5** and **6**). While evidence suggests that TFIIF fits into the following fixed assembly pathway (including simultaneous recruitment with pol II) [3]: TBP → TFIIB → pol II → TFIIF → TFIIH [1],[3] the literature reports seeming conflicting evidence for TFIIE entry [1],[8],[30], and thus we chose to pursue to two alternative assembly mechanisms: TBP → TFIIB → pol II→ TFIIF → TFIIE → TFIIH (**Figure 5**) and one where TFIIE enters prior to pol II (**Figure 6**). We focused on the few clusters that had the most members and had multiple factors with high occupancy (indicated by asterisks). These included clusters with 687, 580, and 252 members (**Figs. 4**, **5**, and **6**). The membership for these particular clusters remained unchanged as more factors were included in the modeling because they failed to generate new gene groups that had sufficient membership to avoid consolidation. Thus, the occupancy data and the associated mechanisms displayed robust consistency as multiple GTF's were added on, which is consistent with them working together in a PIC.

The occupancy levels in the five-factor modeling were compatible with mechanisms that had TBP and pol II dissociate early and TFIIB and TFIIF dissociating late (**Figure 4B**). Interestingly, groups with few genes tended to have a larger number of compatible mechanisms (more green boxes in **Figure 4A**). While the significance of this is unclear, it might reflect a cellular design that avoids ambiguity in the PIC disassembly pathway. That is multiple, alternative dissociation pathways may be problematic to control.

In modeling six factors (**Figure 5**), the predominant compatible disassembly pathways for the two alternative assembly pathways retained the dissociation of TBP and pol II as early steps in all mechanisms. Whether we define TFIIE assembly as early (upper panel) or late (lower panel), the occupancy data supported the following two predominant dissociation mechanisms: P→T→H→B→(E,F) and T→P→(E,F,H)→B, although when E associated early, the following pathway was also acceptable: T→P→(F,H)→(E,B). Spot checks of our results using COPASI confirmed our findings (not shown).

## Discussion

Genome-wide occupancy data for the many hundreds of proteins involved in gene regulation is now accumulating. One major challenge has been to inter-relate such occupancy data and conceptualize it in light of models about how these proteins function together. Such models, as in the case of the assembly of the transcription machinery at promoters, are derived from biochemical experiments conducted on isolated components of the transcription machinery. The extent to which inferred biochemical mechanisms reflect in vivo processes is not known. We are not aware of any means of modeling genome-wide occupancy data to determine whether it is compatible with biochemical mechanisms. To this end, we developed the software tool PathCom. PathCom is generic in that it will determine whether any number of user-defined mechanisms is compatible with measured occupancy data of any number of relevant proteins. We applied PathCom to transcription complex assembly/disassembly, which has been extensively defined biochemically and for which genome-wide ChIP-chip occupancy data is available for. Biological insight gleaned from the modeling is subject to the veracity of the assumptions regarding what in vivo ChIP occupancy data actually measures, and the quality of the data being modeled.

Eukaryotic protein coding genes utilize a common set of general transcription factors to assemble RNA polymerase II at promoters. A long-standing question that biochemistry has attempted to explain is the order of assembly of the transcription machinery and what happens to individual components during multiple transcription cycles. As far as the general transcription machinery is concerned, in vitro ordered assembly starts with TBP followed by TFIIB, then pol II and TFIIF, and then TFIIE and TFIIH [1],[3]. In vivo ChIP occupancy data alone cannot discern whether such an assembly pathway is correct at any or all genes, and thus is a premise of the modeling example employed here. In the context of such a fixed assembly pathway, we explored different occupancy patterns of the general transcription machinery observed across the yeast genome, and interpret such occupancy patterns to potentially reflect alternative dissociation mechanisms. Should alternative association mechanisms be considered, then alternative dissociation mechanisms are likely.

In regards to the genome-wide distribution of the GTF's, we did not see a random partitioning of genes into high vs low occupancy states for each factor. Principal component analysis (PCA) indicates the presence of a single major component (not shown), and several minor ones. This would be consistent with the strong tendency of the GTF's to work together. What is interesting about the PCA is that TFIIB, pol II, TFIIF, and TFIIH were the main drivers in the first principal component, despite pol II having relatively low occupancy at the promoter region. TBP contributed the least to the principal components (**Dataset S1** and **Figure S5**). In addition, we determined whether genes with <10% occupancy or ≥10% occupancy had a tendency toward having TATA versus TATA-less promoters, using data from [31]. We found that approximately 20% of genes with <10% or ≥10% occupancy levels were TATA-containing genes. Therefore, neither group had a bias toward TATA or TATA-less genes. Also we took the very highest TBP binding genes (at least 50% binding) and they also had 20% TATA-box genes. It does not seem likely that factor percent binding shows any correlation with the percent of genes that have TATA-boxes or sequence-effects in general.

When clustering all GTF's and pol II, three high occupancy states stood out as having a large membership. These included genes with high levels of 1) all GTF's, 2) all GTF's except TBP, and 3) all GTF's except TBP and pol II. The group having high levels of all GTF's was by far the most highly transcribed, which is not surprising. This group included the ribosomal protein genes. However, for the major groups, low levels of TBP were more closely linked to low levels of transcription than the occupancy level of any of the other factors including pol II. This confirms on a genomic scale the earlier notion established on a few genes that TBP recruitment or retention is rate-limiting in transcription [32]. However, since pol II and the other GTF's are commonly found at high levels at many promoters even when TBP levels are low, it also seems likely that steps after TBP recruitment will be rate-limiting at certain genes. Otherwise, a rapid initiation and elongation phase would be expected to result in low pol II occupancy at all promoters.

While the number of dissociation mechanisms scale factorially (n!) with the number (n) of proteins involved, we did not see an equal distribution of genes into each type of mechanism, and we did not see a corresponding increase in the number of compatible dissociation mechanisms. Instead, the number of compatible mechanisms remained rather fixed at one to two, for a given association mechanism. The general pattern observed for most genes, was that if TBP, TFIIB, pol II, and the other GTFs assembled in the listed order, then the dissociation order was generally TBP, then pol II, then the other GTFs, with the latter being less resolved.

## Methods

### Occupancy and grouping of genes

#### Background normalization

Factor occupancy data was obtained from ArrayExpress (http://www.ebi.ac.uk/arrayexpress/) using the accession numbers E-MEXP-1676 and E-MEXP-1677 (low-density tiling microarray probes). High-density tiling microarray data was obtained from material in [14]. The 25°C YPD media occupancy data for the TSS probe (in the low-density data) was used for modeling, representing occupancy values near the TSS for 5743 genes. The probe for the TSS was designed for regions between 30 bp and 90 bp upstream from the start of the actual ORF. However, all raw data (from ∼20,000 probes) was processed as follows: First, a background dataset was calculated. Each BY4741 background dataset was normalized to the median value of the entire dataset. Then replicates were combined by computing the median value for each probe. Second, each factor ChIP dataset was divided by the background BY4741 dataset, on a probe-by-probe basis, then divided by the median value for all probe signals located in T-T regions (“tail-to-tail” intergenic regions between convergently transcribed genes, which are expected to be devoid of bound factors). The resulting occupancy levels represent fold over background, centered so that the ratio in nonpromoter regions equals 1. For further information on the experimental design, see [14].

#### Scaling datasets from 0 to 100% occupancy

This scaling was necessary to compare occupancy levels across different factor datasets. In principle such scaling eliminates differences in crosslinking efficiencies and ChIP yields between factors. Fold-over-background values equal to or less than 1 represent background and thus were re-coded as 0% occupancy. Several limitations of the ChIP assay precluded accurate assessment of 100% occupancy. First, ChIP hybridization signals generally correlated with actual occupancy levels but were not tightly linked (see below), and so the maximum detected fold enrichment over background could not simply be set to 100%, inasmuch as the variance might be quite substantial. Second, ChIP assays do not measure absolute binding, and so even if the variance were eliminated, we could not be certain that the maximum detected level of binding represented 100% occupancy. Nonetheless, if all factors are held to the same standard, and data from groups of similarly behaving genes are aggregated, then approximations can be made. Therefore, we coded any value above the 99^th^ percentile rank (top 200 probes) as 100% (setting the 100% mark to the upper 98^th^ percentile gave essentially the same results). All remaining data were scaled between 0 and 100% occupancy by subtracting background (1.0) from all data, and dividing through by the value at the 99^th^ percentile rank.

#### Assumption of linearity of occupancy levels

It is generally assumed that ChIP signals scale linearly with actual occupancy level. However, it is possible that a factor bound to one type of DNA sequence may crosslink more readily than when bound to a different sequence. To test the effect of underlying DNA sequence on crosslinking efficiency, we examined the distribution of TBP occupancy levels at each of the eight TATA box subtypes [31], TATA(A/T)A(A/T)(A/G). As presented in **Dataset S2**, a chi-square test demonstrated that TBP occupancy levels were independent of DNA sequence (p-value  = 0.48). Next we tested TFIIB, which binds both TBP and DNA, and also found it to be independent of the sequence of the TATA box (p-value  = 0.76). Nevertheless, to minimize the influence of crosslinking efficiency on measured occupancy levels, similarly behaving genes were grouped, and their median occupancy level was used in the modeling. In addition, we focused on those groups having high gene membership, which should further alleviate fluctuations associated with individual genes.

#### Grouping of genes into low and high occupancy levels

To increase the robustness of the occupancy values, as well as focus the modeling on predominant patterns, we grouped genes in accordance with their occupancy level for each factor. Genes (probes) having a GTF occupancy below 10% were parsed into low (L) occupancy groups. All others were parsed into high (H) occupancy groups, resulting in 2^n^ theoretically possible groups, where “n” is the number of GTFs being modeled. Parsing the data at a 15% cutoff, or into three groups (low, medium, high using the 10% and 20% for the low-medium and medium-high cutoffs, respectively) did not substantially alter the outcomes, and its main conclusions.

Groups having low membership do not represent predominant patterns and so were consolidated as follows: Groups having >100 genes were exempt from consolidation because they have substantial membership, and groups having <10 genes were required to be consolidated for lack of viable membership. Otherwise, if the membership of an existing group was split by more than a 4∶1 ratio when an additional factor was added to the model (e.g. from 2-factor models to 3-factor models), then the two resulting clusters were consolidated (i.e., not split; note that the label of the consolidated clusters was assigned the label of the larger cluster). The final occupancy median calculations can be found in **Dataset S3**. Because of consolidation, the number of actual clusters is less than 2^n^. Note that consolidation was not performed when we were analyzing the two- and three-factor models in order to make the modeling explanations more clear.

### PathCom

PathCom requires the user to enter occupancies of proteins in a tab-delimited text file followed by the name of the cluster line by line. In a header, before the occupancies are entered, users enter one-letter codes to denote protein identities (of the user's choice) followed by a number to indicate the order in which the proteins assemble (See **Text S1** for information how PathCom was designed and how it was intended to be used). Below each protein in the header, the user enters the percent occupancies calculated along with the name of each cluster (or gene). After execution, the program then reads each cluster's occupancies on each line. Given the fixed order of association of proteins specified by the user in the header, the program generates all possible dissociation sequences. Note that if the user changes the association order, the pool of dissociation reactions will remain the same, but the numbering of each dissociation reaction will be different, because PathCom uses the specific association to generate the dissociation sequences. The program processes each dissociation sequence, pairing it with the fixed association sequence, and given the rules of compatibility (discussed in the paper), computes whether the input protein occupancies are compatible with the mechanism (association + dissociation) it is testing. PathCom processes all possible dissociation sequences for all groups entered. PathCom writes the results to a tab-delimited text file. In this file, the horizontal axis is labeled with every mechanism identification number and the vertical axis is labeled with every cluster name. Also, PathCom writes a file that matches each dissociation sequence with its dissociation sequence identification number. Every time a set of occupancies and a mechanism are compatible, the program reports “−1”, and when they are not, the program reports “0.” Results can be clustered through Cluster then visualized graphically in Treeview [33] The code is given in **Protocol S1 and S2** for users of Windows and Mac OS, respectively.

### COPASI

COPASI conducts chemical kinetic and stochastic simulations [9], and is freely available for download at www.copasi.org. Reactions were set to be irreversible for simplicity. Initial input protein and DNA concentrations were set to be equal, having an arbitrary value of 10 (setting the DNA concentration to 1 gave the exact same results in terms of compatibility, **Figure S6**). Since the observed occupancy levels for a factor represent the sum of all intermediate species having that factor, it was necessary to employ the Parameter Estimation function to optimize this sum, using the free protein concentration equal to (1 – Occ/100)×10, where “Occ” is the measured percent occupancy level, and had a practical lower limit of 0.1% (this formula is only valid when all species concentrations were set to 10). The Parameter Estimator may converge on a local minimum, which may not represent the optimal solution. Running the estimator multiple times alleviated the local minimum, since it employs a random search component. COPASI creates an objective value (E) used to measure goodness of fit between simulated and measured values:
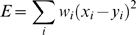
where “i” represents each of the protein factors involved in the modeling, “w” is the weight that is given to a particular protein in the optimization procedure, which is calculated automatically by COPASI, “x” is the measured occupancy, and “y” is the simulated occupancy. Since COPASI aims to minimize this sum of squares, lower E values (more negative log_10_ E) reflect better congruence between modeled and measured data.

Since each modeling run has a manual component and becomes computationally draining with a large number of factors, it became impractical to run COPASI to fully generate the compatibility charts for four or more factors. Nonetheless, we employed COPASI to spot check these charts, and found 100% agreement with PathCom.

## Supporting Information

Figure S1 Scatter plot showing the distribution of percent of maximally measured occupancy of TBP and TFIIB.
(0.16 MB TIF)

Figure S2 Scatter plots showing the occupancy level of each replicate. Also shown are two plots comparing the median percent occupancies of TBP and TFIIB in the four two-factor clusters using both the low and high density tiling array data.
(0.26 MB TIF)

Figure S3 All six possible three-factor assembly pathways are shown and their corresponding PathCom compatibility cluster plots are shown, detailing which possible disassembly pathways arise under each possible assembly pathway. See Figure 2A to see which numbers correspond to which disassembly mechanisms.
(0.23 MB TIF)

Figure S4 Shown are the experimentally determined digital footprints of genes having the highest occupancy of TBP (with TATA-boxes). The bases boxed in red highlight the TATA-boxes. The lack of discernable footprints suggests that TBP does not fully occupy its most occupied sites.
(0.28 MB TIF)

Figure S5 The two strongest principal components in a Principal Components Analysis (PCA) done on the six general transcription factors. They are plotted to show each factor's relative contribution to the principal components.
(0.11 MB TIF)

Figure S6 Compatibility chart for three factor modeling using COPASI, in which the DNA concentration was reduced from 10 to 1.
(0.20 MB TIF)

Protocol S1 PathCom code for Windows users.
(0.01 MB TXT)

Protocol S2 PathCom code for Mac OSX users.
(0.01 MB TXT)

Text S1 Instruction on how to use PathCom.
(0.52 MB DOC)

Dataset S1 Principal Component Analysis (PCA) of the six GTF's
(0.01 MB XLS)

Dataset S2 The results of chi-square testes on whether underlying TATA-sequence variation might have had any effect on the cross-linking efficiencies of TBP and TFIIB.
(0.04 MB XLS)

Dataset S3 Median occupancy levels for gene groups
(0.03 MB XLS)
