# Supplementary figures and images for: Genome-Wide Modeling of Transcription Preinitiation Complex Disassembly Mechanisms using ChIP-chip Data
Source: PLoS Comput Biol. 2010 Apr 1;6(4):e1000733. doi: 10.1371/journal.pcbi.1000733 (PMC2848545; doi:10.1371/journal.pcbi.1000733)

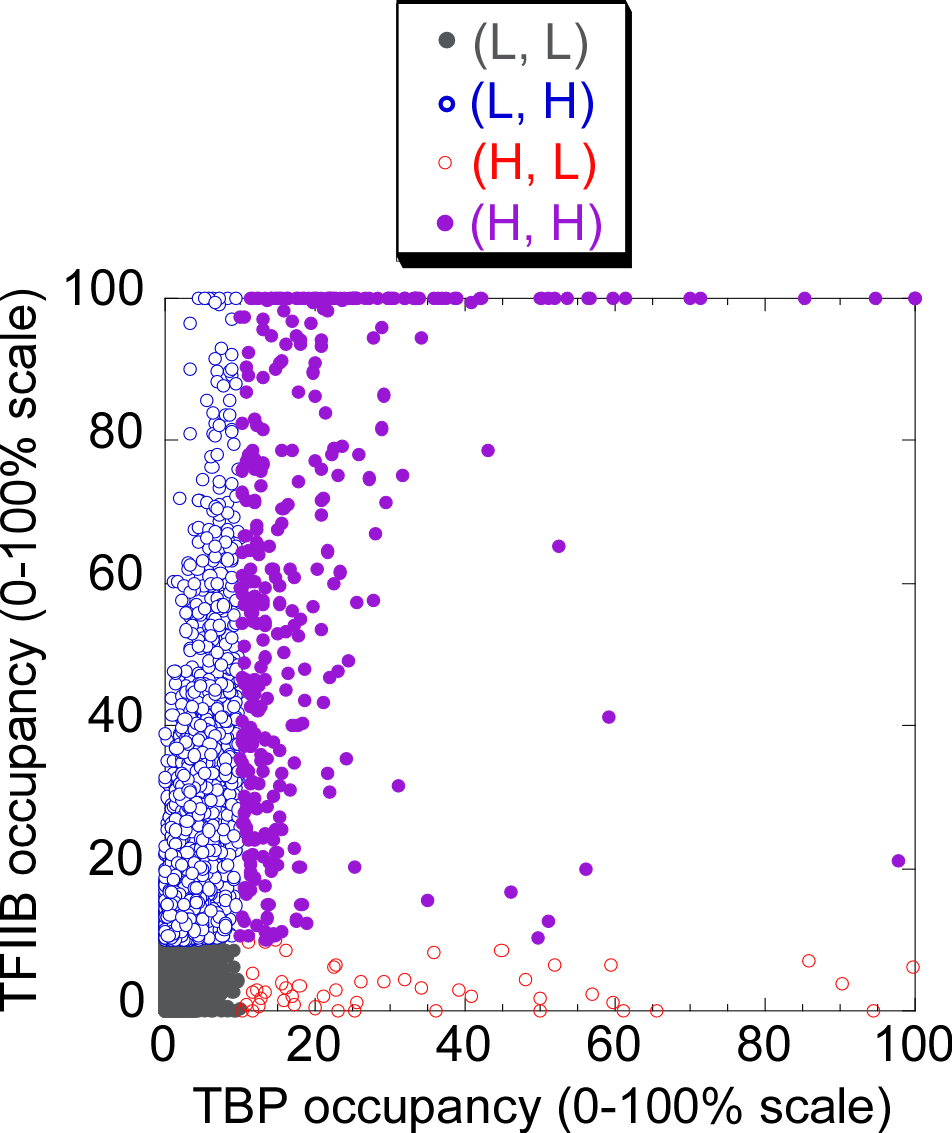

Supplement: Figure S1 — Scatter plot showing the distribution of percent of maximally measured occupancy of TBP and TFIIB. (0.16 MB TIF) [file pcbi.1000733.s001.tif]

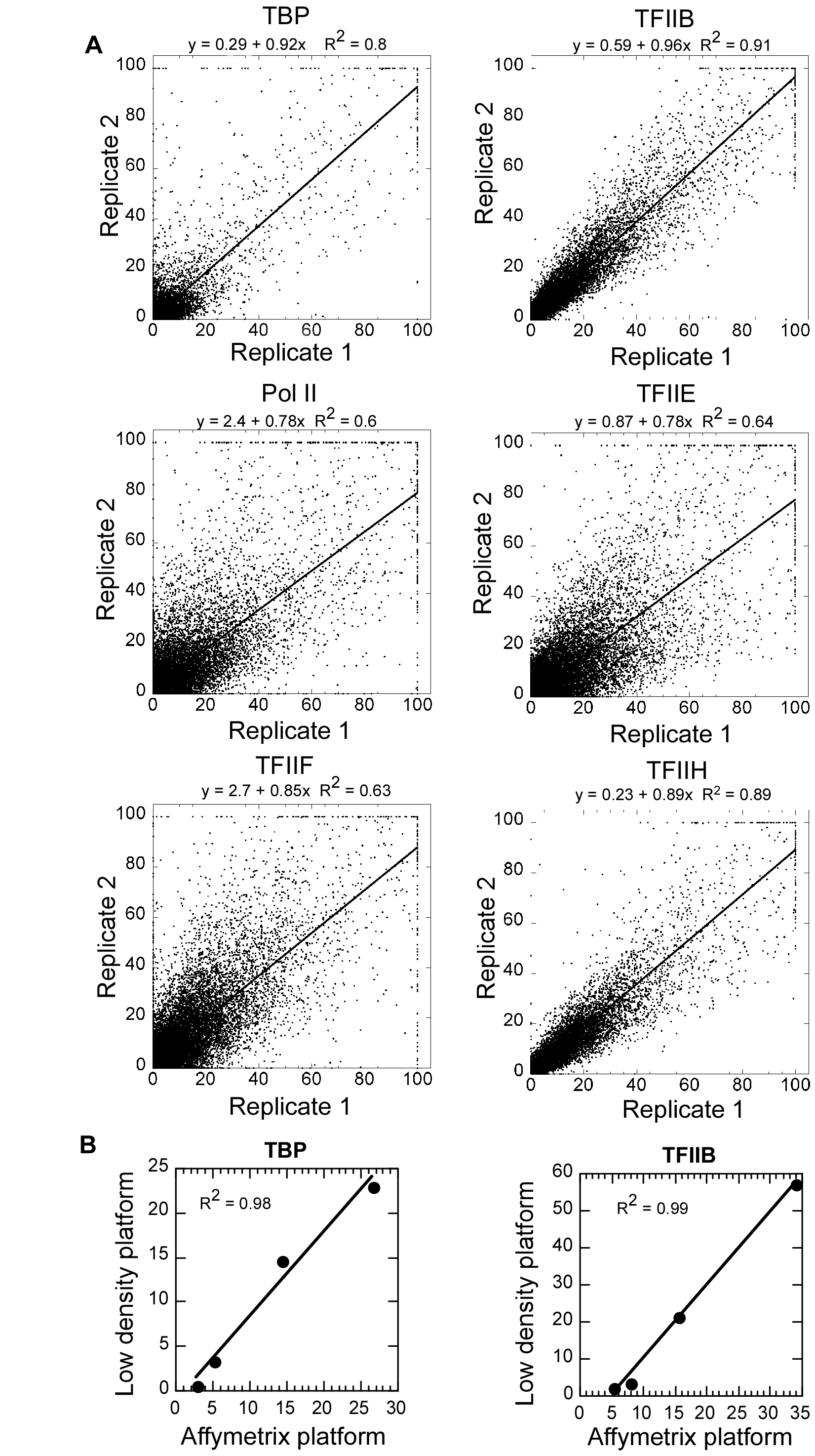

Supplement: Figure S2 — Scatter plots showing the occupancy level of each replicate. Also shown are two plots comparing the median percent occupancies of TBP and TFIIB in the four two-factor clusters using both the low and high density tiling array data. (0.26 MB TIF) [file pcbi.1000733.s002.tif]

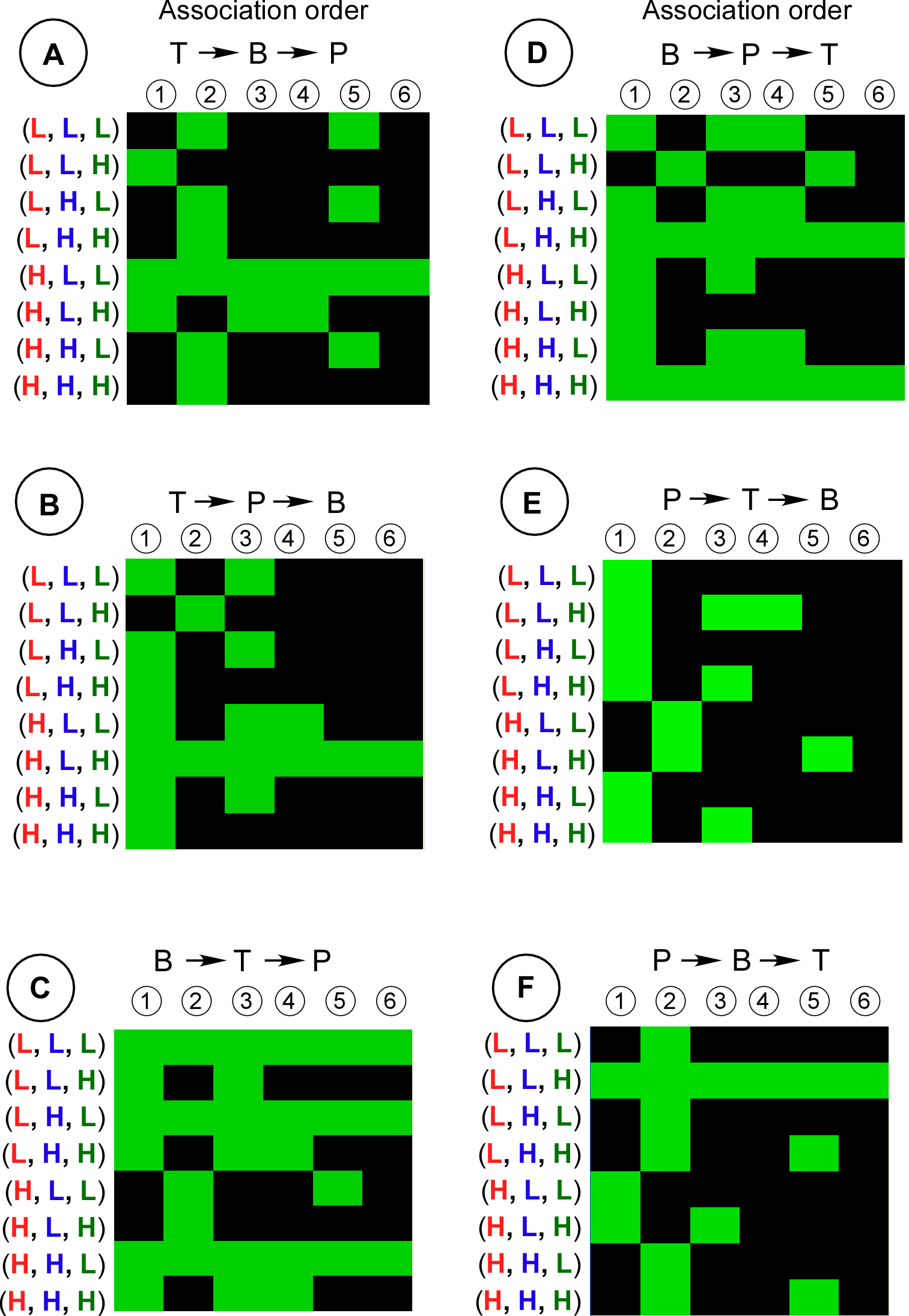

Supplement: Figure S3 — All six possible three-factor assembly pathways are shown and their corresponding PathCom compatibility cluster plots are shown, detailing which possible disassembly pathways arise under each possible assembly pathway. See Figure 2A to see which numbers correspond to which disassembly mechanisms. (0.23 MB TIF) [file pcbi.1000733.s003.tif]

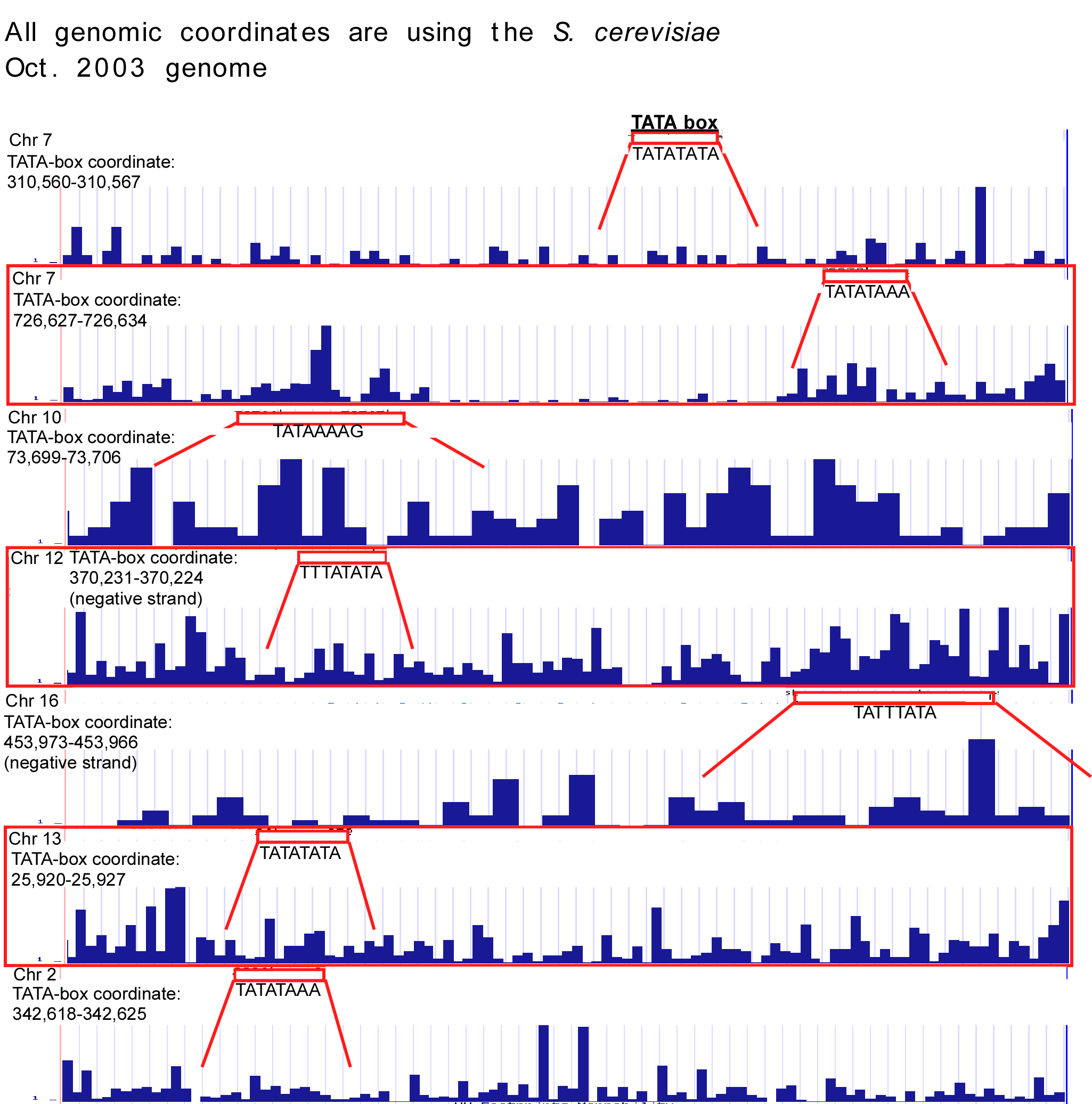

Supplement: Figure S4 — Shown are the experimentally determined digital footprints of genes having the highest occupancy of TBP (with TATA-boxes). The bases boxed in red highlight the TATA-boxes. The lack of discernable footprints suggests that TBP does not fully occupy its most occupied sites. (0.28 MB TIF) [file pcbi.1000733.s004.tif]

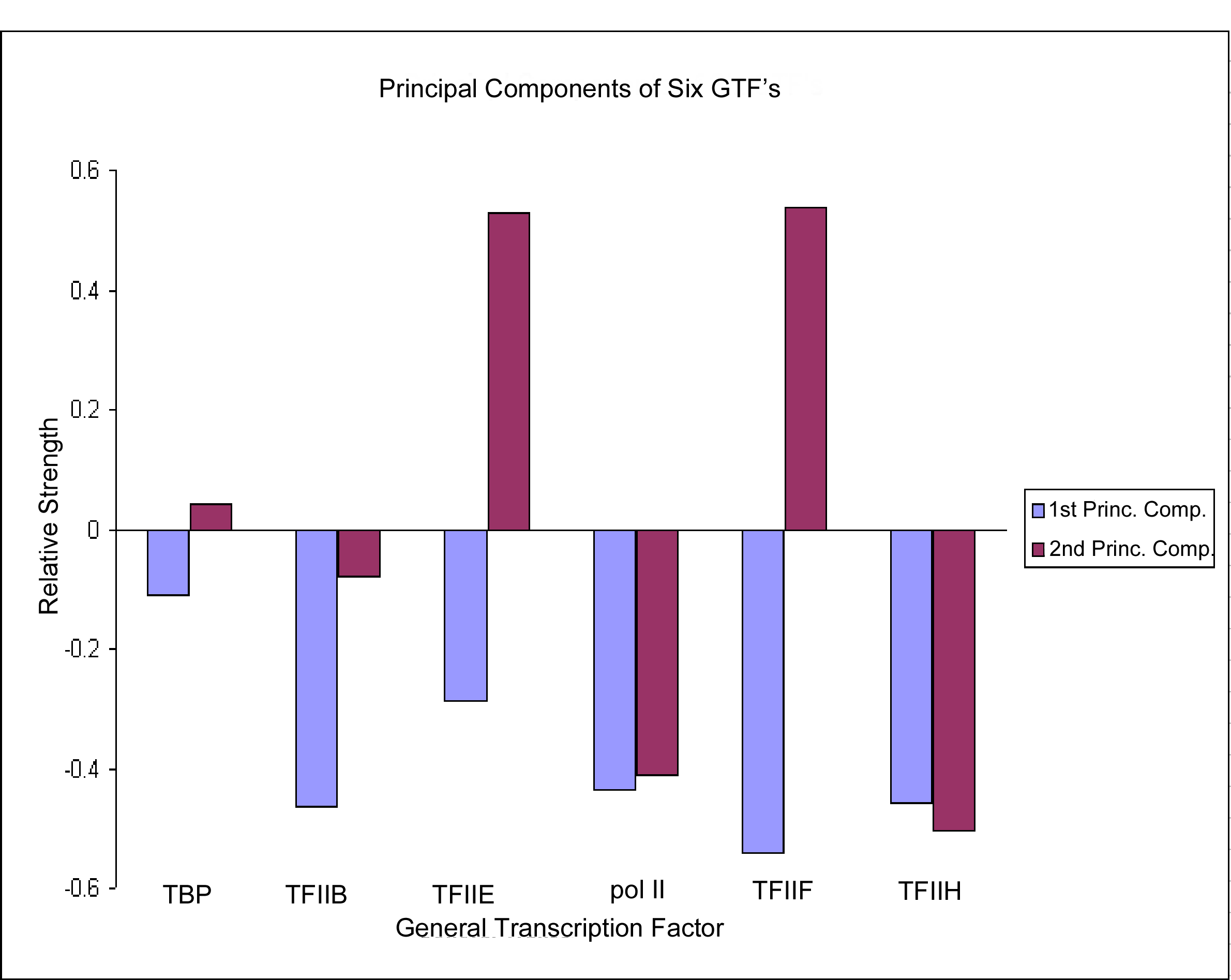

Supplement: Figure S5 — The two strongest principal components in a Principal Components Analysis (PCA) done on the six general transcription factors. They are plotted to show each factor's relative contribution to the principal components. (0.11 MB TIF) [file pcbi.1000733.s005.tif]

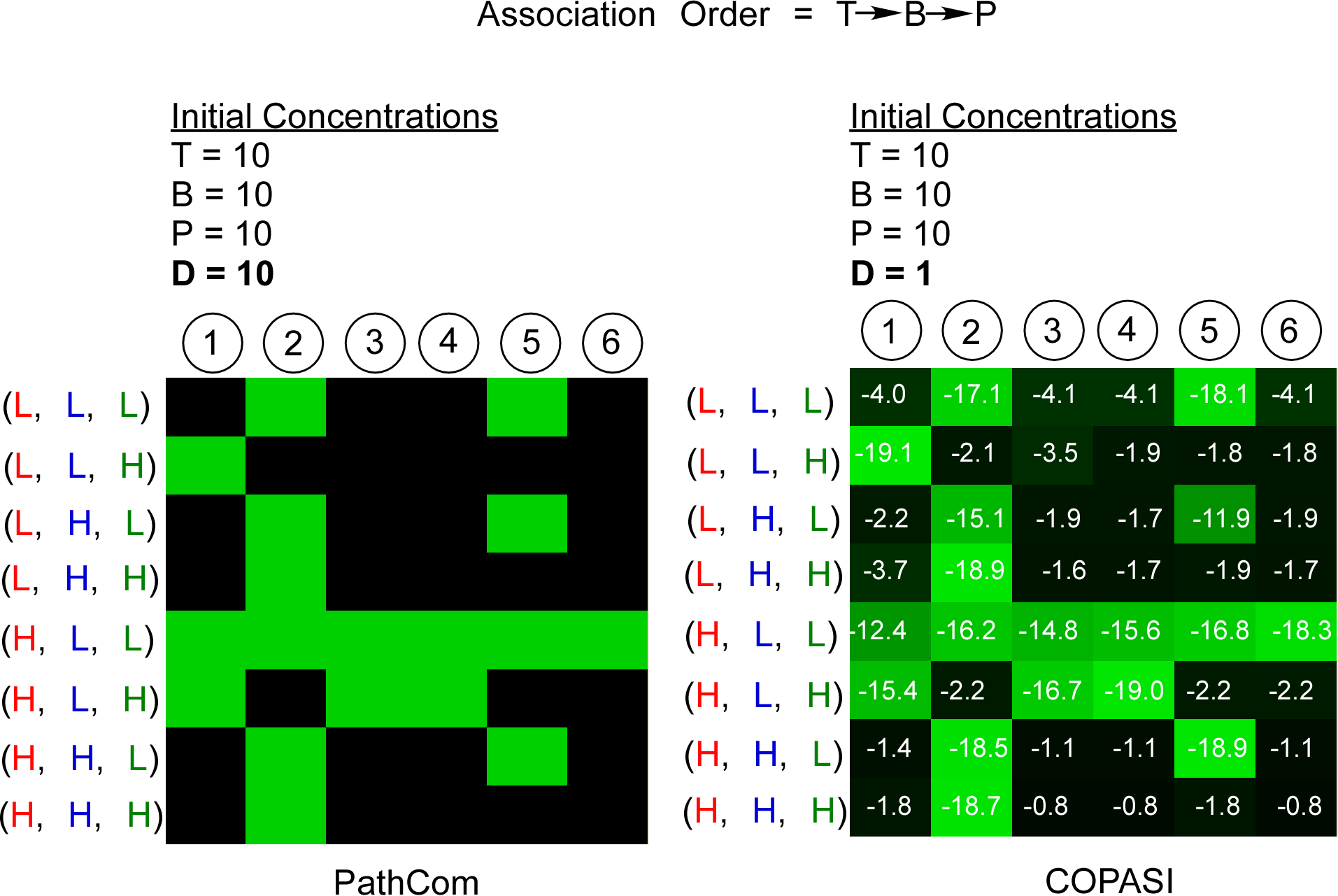

Supplement: Figure S6 — Compatibility chart for three factor modeling using COPASI, in which the DNA concentration was reduced from 10 to 1. (0.20 MB TIF) [file pcbi.1000733.s006.tif]
