# Supplementary material for: Genome-Wide Modeling of Transcription Preinitiation Complex Disassembly Mechanisms using ChIP-chip Data
Source: PLoS Comput Biol. 2010 Apr 1;6(4):e1000733. doi: 10.1371/journal.pcbi.1000733 (PMC2848545; doi:10.1371/journal.pcbi.1000733)
Supplement: Text S1 — Instruction on how to use PathCom. (0.52 MB DOC) [file pcbi.1000733.s009.doc]

**General Overview**

The idea of the PathCom, the program written here, is to be able to take percent occupancies and the PIC association reaction the user enters and then generate all the possible dissociation reactions and tell which dissociation reactions are compatible with each dataset of percent occupancies that are entered. One can enter a dissociation reaction and the program will the user the compatible association reactions, just if one wants to enter a dissociation reaction, one has to enter it in reverse.

**Installation**

There are some libraries we needed to download in order to provide all the necessary functions for the program to work. First the programming language Python had to be installed on the computer on which the analysis was done. We went to <http://www.python.org/> to install the language on our computer. We also had to install the Matplotlib library, which can be found at <http://matplotlib.sourceforge.net/>. We also used the Numpy library, which can be found at <http://sourceforge.net/projects/numpy/files/>. We made sure the versions of Matplotlib and Numpy are for the correct our operating system, which, for our case was, a PC. We also made sure that these libraries were for the correct version of Python.

**The workings of PathCom**

The percent occupancies we computed were entered into the text file Text2.txt. This file needs to be tab-delimited in order to work. To enter the occupancies, we found it easiest to open this text file in Excel, which after we entered the occupancies in individual cells, will automatically convert these cells into the tab-delimited file.

In the text file Text2.txt (if the input file is not called Text2.txt, the program will not run), the first line is the header where we entered the proteins that we wished to model (Figure 1, the first line). Because of the way the program is designed, we had to use one-letter abbreviations for all the proteins we wanted to model followed by the number indicating the order each protein comes in (Figure 1). **Note: The names “Protocol_S1” and “Protocol_S2” have underscores, because Python will not run with spaces in the names. Therefore, if the name of Python code is changed, make sure that the new name has spaces in it.**

Figure 1- The first line is the header where all the one letter codes are used to indicate proteins in PIC assembly followed by the order they come in are listed (cells A1 and A2, which indicate ”T” comes in first and “B” comes in second) along with the “Cluster” label (cell A3). Below the header, the protein percent occupancies for each cluster are listed. Under the “Cluster” header, the names of all the clusters are written. Note that “Text2.txt” is written at the top of the figure, but it is opened in Excel.

Below the header, we entered the percent occupancies. Also, for proper delineation, we programmed PathCom to make sure the cluster names are explicit, there we make the user type “Cluster” in the top line. For the example, in Figure 1, the first line should go “T1” , “B2”, and finally “Cluster” to show that, in this example, TBP and TFIIB are being modeled with TBP coming in first and TFIIB coming in second. Underneath the header, we wrote the percent occupancies for T and B and the cluster names.

After all the aforementioned inputs were entered, we had to close the text (and make sure that it was saved correctly, one can open it as a text file to make certain everything was saved correctly), we executed the code. It is easiest to execute this code from your Command Prompt.

To output, the program will write another tab-delimited text file CompatibilityTable.txt (which we also opened in Excel for easy visualization). In this text file, there will be a table where each row will be labeled with each cluster label that PathCom found in Text2.txt. Each column will be labeled with each mechanism number. Where there is compatibility, PathCom will print "-1" to the appropriate row and column, where there is incompatibility, PathCom will print "0" (Figure 2)

Figure 2 Results of running the program for compatibility testing with two factors. A “-1” indicates compatibility and a “0” indicates incompatibility.

In order to produce the black-green compatibility tables shown in the paper, we took the text file CompatibilityTable.txt and uploaded it into the program Cluster [1] (before we did that, we had to open CompatibilityTable.txt in Excel. **In cell A1, we retyped “Cluster” and hit tab and saved and closed the file**. This is a small squak to be fixed in order to make the output compatible with the program Cluster). When we opened CompatibilityTable.txt in Cluster, we had to make sure the number of rows and columns indicated were correct. In the program Cluster, one can hierarchically or K-means cluster the data the way one would with gene expression and ChIP-occupancy data. The results of this were then viewed in the program TreeView [1], which was used to generate the black-green matrices in the report. For convenience, we also programmed PathCom to write another text file “Dissociations.txt,” that shows which mechanism numbers correspond to which dissociation orders.

Note that text and Excel files cannot display more than 256 columns, which means one cannot run more than 5 factors on this (CompatibilityTable.txt won’t display all the results). If one intends on running more than 5 factors at once, if one can understand how the code works, one can go into the code and split up the task of running the compatibility testing into different pieces, so that no more than 255 dissociation mechanisms are run at once. Otherwise, one might to split the association reaction up into a few pieces to make them smaller.

References

1. Eisen MB, Spellman PT, Brown PO, Botstein D (1998) Cluster analysis and display of genome-wide expression patterns. Proc Natl Acad Sci U S A 95: 14863-14868.
